# The genetic relationships between immune cell traits, circulating inflammatory proteins and preeclampsia/eclampsia

**DOI:** 10.3389/fimmu.2024.1389843

**Published:** 2024-05-30

**Authors:** Yu Liu, Yuliang Zhang, Lili Du, Dunjin Chen

**Affiliations:** ^1^ Department of Obstetrics and Gynecology, Guangdong Provincial Key Laboratory of Major Obstetric Diseases, The Third Affiliated Hospital of Guangzhou Medical University, Guangzhou, China, Guangzhou, Guangdong, China; ^2^ Guangdong-Hong Kong Macao Greater Bay Area Higher Education Joint Laboratory of Maternal-Fetal Medicine, The Third Affiliated Hospital of Guangzhou Medical University, Guangzhou, China, Guangzhou, Guangdong, China

**Keywords:** preeclampsia/eclampsia, immune cell phenotypes, circulating inflammatory proteins, Mendelian randomization (MR) analaysis, genetic epidemiology

## Abstract

**Objectives:**

Preeclampsia/eclampsia (PE), a critical complication during pregnancy, has been suggested to correlate with immune cell phenotypes and levels of circulating inflammatory proteins. Our study aimed to employ a two-sample mendelian randomization (MR) analysis to assess the potential causal effects of immune cell phenotypes and circulating inflammatory proteins on the onset of PE.

**Methods:**

We utilized summary-level data from genome-wide association studies (GWAS). This included statistics for 371 immune cell phenotypes from 3,757 individuals in the Sardinian founder population, and data on 91 circulating inflammatory proteins from 14,824 European ancestry participants. Additionally, genetic associations related to PE were extracted from the FinnGen consortium, involving 1,413 cases and 287,137 controls. We applied inverse variance weighting (IVW) and supplementary methods like MR-Egger, weighted median, and weighted mode to comprehensively assess potential causal links.

**Results:**

Our analysis revealed significant causal associations of several immune cells type and inflammatory proteins with PE. Out of the immune cell phenotypes analyzed, six immune phenotypes emerged as significant risk factors (*p <*0.01), mainly include CD4 on activated and secreting CD4 regulatory T cells, CD28 on CD39+ CD4+ T cells, CD127- CD8+ T cell absolute cell (AC) counts, HLA DR on HLA DR+ CD8+ T cell, CD66b on CD66b++ myeloid cells, and HLA DR on dendritic cells. And ten were identified as protective factors (*p <*0.01). Such as CD45 on CD33br HLA DR+ CD14-, CD33+ HLA DR+ AC, CD33+ HLA DR+ CD14- AC, CD33+ HLA DR+ CD14dim AC, CD27 on CD24+ CD27+ B cell, CD20- CD38- %B cell, IgD- CD24- %B cell CD80 on plasmacytoid DC, CD25 on CD4+ T cell, and CD25 on activated & secreting CD4 regulatory T cell. Furthermore, among the inflammatory proteins studied, five showed a significant association with PE, with three offering protective effects mainly include that C-X-C motif chemokine 1, tumor necrosis factor ligand superfamily member 14, and C-C motif chemokine 19 and two exacerbating PE risk such as STAM-binding domain and Interleukin-6 (p <0.05).

**Conclusions:**

Our study highlights the pivotal roles played by diverse immune cell phenotypes and circulating inflammatory proteins in the pathophysiology of PE. These findings illuminate the underlying genetic mechanisms, emphasizing the criticality of immune regulation during pregnancy. Such insights could pave the way for novel intervention strategies in managing PE, potentially enhancing maternal and neonatal health outcomes.

## Introduction

Preeclampsia/eclampsia (PE) represent prevalent complications during pregnancy, affecting an estimated 3–8% of pregnancies globally, with an increasing incidence over time (1). These disorders are not only prevalent but also contribute significantly to maternal and neonatal morbidity and mortality. The complexities surrounding the complications, injuries, long-term prognosis, and sequelae for both the mother and child following pregnancy termination underscore the unpredictability and severity of these conditions (2, 3). PE imposes a substantial burden, extending its impact from the affected individuals to public healthcare systems and family units, with pronounced effects in low- and middle-income countries. Addressing the incidence and mortality of pregnancy-related hypertensive disorders is, therefore, a pressing global health priority, with implications for maternal and infant well-being.

Research in recent decades has intensively investigated the pathogenesis of preeclampsia. These studies have uncovered a multitude of mechanisms including endothelial dysfunction, aberrant vascular development, compromised trophoblast invasion, and inadequate remodeling of spiral arteries (4–6). Particularly noteworthy is the role of immune dysregulation and inflammation, which are now understood as key contributors to placental dysfunction, leading to the manifestation of preeclampsia (4, 7, 8). However, aberrations in immune function may contribute to placental ischemia, a key factor in the pathogenesis of PE (9). In addition to immune cells, circulating inflammatory proteins derived from the placental and maternal endothelial systems also play a role. It has been proposed that imbalance of cytokine levels and elevated soluble cytokine receptors in PE patients may serve as markers of inflammation, but these findings are somewhat controversial (10–12). However, most identified factors lack accuracy in predicting the onset of PE. This emphasizes the need for further investigation into the factors associated with PE and interventions aimed at prolonging gestation and improving maternal and neonatal outcomes. However, the diversity in research findings concerning the relationship between immune cells, inflammatory factors, and PE points to inherent challenges in this field. These include the alignment of PE models with human physiological states, constraints in sample sizes, design flaws in studies, and the presence of confounding variables. Addressing these challenges, mendelian randomization (MR) presents an innovative method, which leverages genetic variants as instrumental variables to estimate the causal association between exposure and disease outcome (13). These variants, determined at conception and randomly assigned, enable two-sample MR to act as an unbiased method for examining the impact of exposures on disease outcomes, thus overcoming the limitations of confounding and reverse causation (14, 15). MR has found application in investigating causal associations between immune cells and various diseases, encompassing immune disorders (16), cardiovascular diseases (17) and metabolic diseases such as type 2 diabetes (18). In our study, we apply two-sample MR analysis to deepen our understanding of the causal implications of peripheral immunity in the risk of PE, providing new insights into potential therapeutic strategies and preventive measures.

## Materials and methods

### Study design

**Figure 1:**
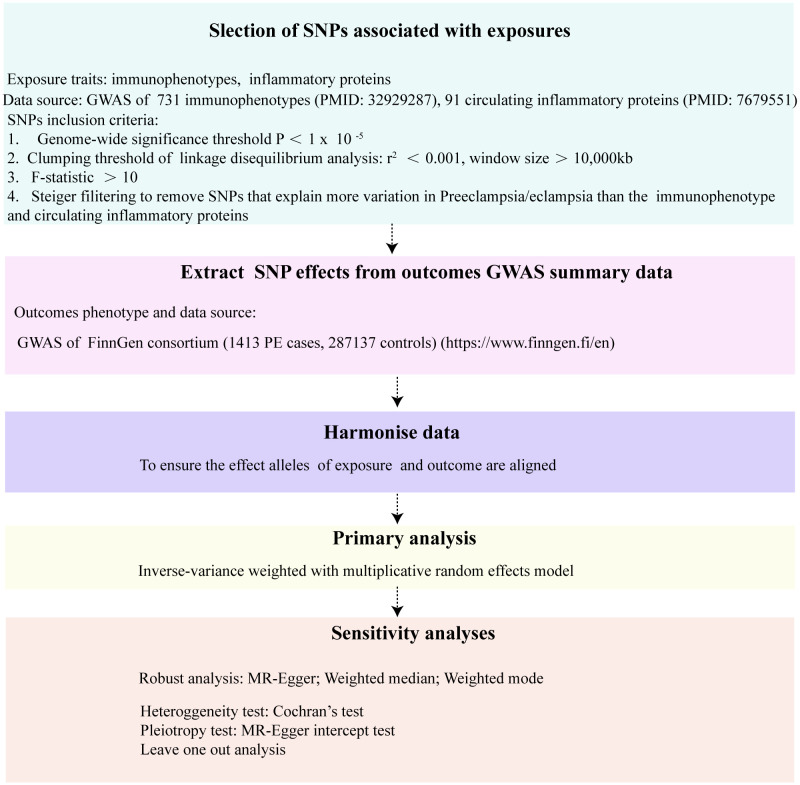
The flowchart of the Mendelian randomization study that the causal association between 731 immunophenotypes, 91 inflammatory proteins and PE.

Our investigation rigorously examined the causal relationships between an array of 731 immune cell phenotypes, 91 circulating inflammatory proteins, and the incidence of preeclampsia/eclampsia (PE). The study’s design strictly followed the STROBE-MR (Strengthening the Reporting of Observational Studies in Epidemiology using Mendelian Randomization) guidelines (19). The integrity and robustness of two-step Mendelian Randomization (MR) outcomes hinge upon the satisfaction of three pivotal assumptions. Firstly, the Relevance Assumption posits that genetic variants utilized as instrumental variables must demonstrate a significant and robust association with the exposure under investigation. This foundational premise ensures that the genetic instruments are effectively linked to the exposure, thereby facilitating a valid examination of the exposure-outcome relationship. Secondly, the Independence Assumption mandates that these genetic variants remain uninfluenced by any confounders, underpinning the concept that the allocation of genetic variants occurs in a manner akin to random assignment at conception, thus safeguarding against confounding biases. Lastly, the Exclusion Restriction necessitates that the influence of genetic variants on the outcome is mediated exclusively through the exposure, precluding any alternative pathways. This assumption is critical for attributing any observed association directly to the exposure of interest, thereby reinforcing the causal inference drawn from the MR analysis. Selection of genetic instrumental variables (IVs), specifically, single-nucleotide polymorphisms (SNPs) was meticulously based on three critical criteria: robust association with immune phenotypes or inflammatory proteins, absence of links to confounding factors that might influence the exposure-outcome relationship, and a direct influence on the outcomes through the exposures without involvement in alternate pathways. The design and progression of our study are encapsulated in **Figure 1**.

### Exposure GWAS data sources

Our study utilized immunological data from a comprehensive genome-wide association study (GWAS), cataloged with accession numbers ranging from GCST90001391 to GCST90002121, and comprising a diverse spectrum of 731 immunophenotypes. These encompass 118 absolute cell (AC) counts, 389 median fluorescence intensities (MFI), 32 morphological parameters (MP), and 192 relative cell (RC) counts (20). Covering an extensive range of immune cells, this dataset included B cells, dendritic cells (DCs), T cells at various maturity stages, monocytes, myeloid cells, and TBNK (T cells, B cells, natural killer cells) and Treg panels, with MPs specifically focusing on DCs and TBNK panels. The initial GWAS analyzed a dataset that included 3,757 individuals of European descent, with a careful design to avoid any overlap between cohorts. High-density arrays were employed to genotype approximately 22 million SNPs, and subsequently, imputation was performed using a reference panel based on Sardinian sequences. Data on 91 circulating inflammatory proteins were derived from a GWAS conducted on 14,824 participants of European ancestry (21). This study identified multiple common genetic variants that influence the levels of circulating cytokines.

### Selection of instrumental variables

The significance level for selecting IVs for each immune trait was set at 1×10^−5^. The linkage disequilibrium (LD) pruning of these SNPs, where SNPs were pruned based on an LD r^2^ threshold of less than 0.001 within a 10000 kb distance. For the selection of IVs for circulating inflammatory proteins, the significance level was set at 1×10^−5^, consistent with the approach for immune traits. SNPs were pruned based on an LD r^2^ threshold of less than 0.001 within a 10000 kb distance. To address the issue of weak instrumental variables, we estimated the F-statistic for each genetic variant used as an instrument. SNPs with an F-statistic below 10 were considered weak and subsequently excluded from our analysis, ensuring the robustness of our findings. For the outcome PE the significance level for IV selection was adjusted to 5×10^-5^.

Steiger filtering is a statistical method designed to eliminate invalid IVs. The underlying principle of this method is that valid IVs should explain more variance in exposure than outcome traits. As a result, genetic variants that do not meet this criterion are discarded. By retaining genetic variation that explains a larger portion of the variance in exposure traits, Steiger filtering can effectively mitigate potential reverse causality effects (22). To circumvent the potential reverse causality of PE resulting in retinal thinning in this study, we employed the Steiger filtering method to exclude SNPs that explain a greater variance in PE-related traits compared to immune trait and inflammatory proteins.

### Genetic associations for outcomes

The study focused on PE. To investigate the genetic associations linked to these conditions, data was gathered from the FinnGen consortium (https://www.finngen.fi/en). This data was part of their 9th release of GWAS summaries, which was made available on May 11, 2023. The dataset included 1413 cases and 287137 controls.

### Statistical analysis strategy

We employed a two-sample MR analysis to examine the relationships among 731 immune cell phenotypes, 91 circulating inflammatory proteins, and the occurrence of PE. Our primary analysis utilized the inverse variance-weighted (IVW) method, a well-established approach in MR studies (23). To enhance result robustness, we conducted supplementary analyses using the MR-Egger regression methods (24), weighted median (25) and weighted mode (26). We assessed potential directional pleiotropy by examining the intercept value in the MR-Egger regression (27). Heterogeneity was evaluated using Cochran’s Q test (28). All statistical analyses and data visualisation were performed using R software, version 4.3.1. For mendelian randomization, we utilized the”TwoSampleMR”,”MendelianRandomization” package in R, which facilitated dataset harmonization and the implementation of various MR methods.

## Results

### Selection of IVs

After conducting a comprehensive screening for multiple conditions, we identified 468 single nucleotide polymorphisms (SNPs) to serve as instrumental variables (IVs) for the exposure factors (including immune cell traits and inflammatory proteins). The F statistics, used to assess the validity of these IVs for both immune cells and inflammatory proteins, consistently exceeded the threshold of 10. This suggests that the possibility of bias due to weak instruments is low. These results are detailed in **Supplementary Tables S1**, **S2**.

### Overview

In our study, we identified the causal roles of 42 immune cells in the development of PE. Using a p-value of less than 0.01 as a screening criterion, we pinpointed six immunophenotypes as risk factors for PE: CD4 on activated and secreting CD4 regulatory T cells (Treg panel), CD28 on CD39+ CD4+ T cells (Treg panel), CD127- CD8+ T cell AC (Treg panel), HLA DR on HLA DR+ CD8+ T cell (TBNK panel), CD66b on CD66b++ myeloid cells (Myeloid cell panel), and HLA DR on dendritic cells (cDC panel). Conversely, we identified ten immunophenotypes that appear to have protective effects against PE. These include CD45 on CD33br HLA DR+ CD14- (Myeloid cell panel), CD33+ HLA DR+ AC (Myeloid cell panel), CD33+ HLA DR+ CD14- AC (Myeloid cell panel), CD33+ HLA DR+ CD14dim AC (Myeloid cell panel), CD27 on CD24+ CD27+ B cell (B cell panel), CD20- CD38- %B cell (B cell panel), IgD- CD24- %B cell (B cell panel), CD80 on plasmacytoid DC (cDC panel), CD25 on CD4+ T cell (Treg panel), and CD25 on activated & secreting CD4 regulatory T cell (Treg panel).

Furthermore, our study revealed insights into the participation of five circulating inflammatory proteins in PE. Specifically, we observed that heightened levels of C-X-C motif chemokine 1, Tumor necrosis factor ligand superfamily member 14, and C-C motif chemokine 19 act as protective factors against PE. Conversely, elevated levels of STAM-binding protein and Interleukin-6 were identified as risk factors (p <0.05). The results of steiger test indicate that the 16 types of immune cells and 5 circulating inflammatory proteins we identified as having a causal association with PE show steiger test p-values less than 0.05. This suggests that these findings do not have reverse causal relationships. The results are summarized in **Supplementary Tables S3**, **S4**.

### Causal associations between immune cell traits and PE

**Figure 2:**
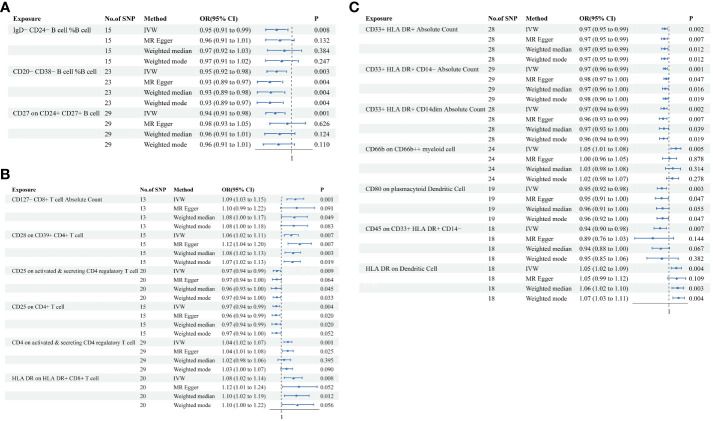
MR analysis illustrating causal links between immune cell phenotypes and PE. This figure presents the results using inverse-variance weighting (IVW), MR Egger, Weighted median and Weighted mode method, displaying odds ratios (OR), confidence intervals (CI), and the relevant single nucleotide polymorphisms (SNPs) associated with the study. **(A)** IgD-CD24- B cell %B cell, CD20-CD38- B cell %B cell, CD27 on CD24+ CD27+ B cell; **(B)** CD127- CD8+ T cell Absolute Count, CD28 on CD39+ CD4+ T cell, CD25 on active & secreting CD4 regulatory T cell, CD25 on CD4+ T cell, CD4 on activated & secreting CD4 regulatory T cell, HLA DR on HLA DR+ CD8+ T cell; **(C)** CD33+ HLA DR+ Absolute Count, CD33+ HLA DR+ CD14- Absolute Count, CD33+ HLA DR+ CD14dim Absolute Count, CD66b on CD66b++ myeliod cell, CD80 on plasmacytoid Dendritic Cell, CD45 on CD33+ HLA DR+ CD14-, HLA DR on Dendritic Cell.

**Figure 3:**
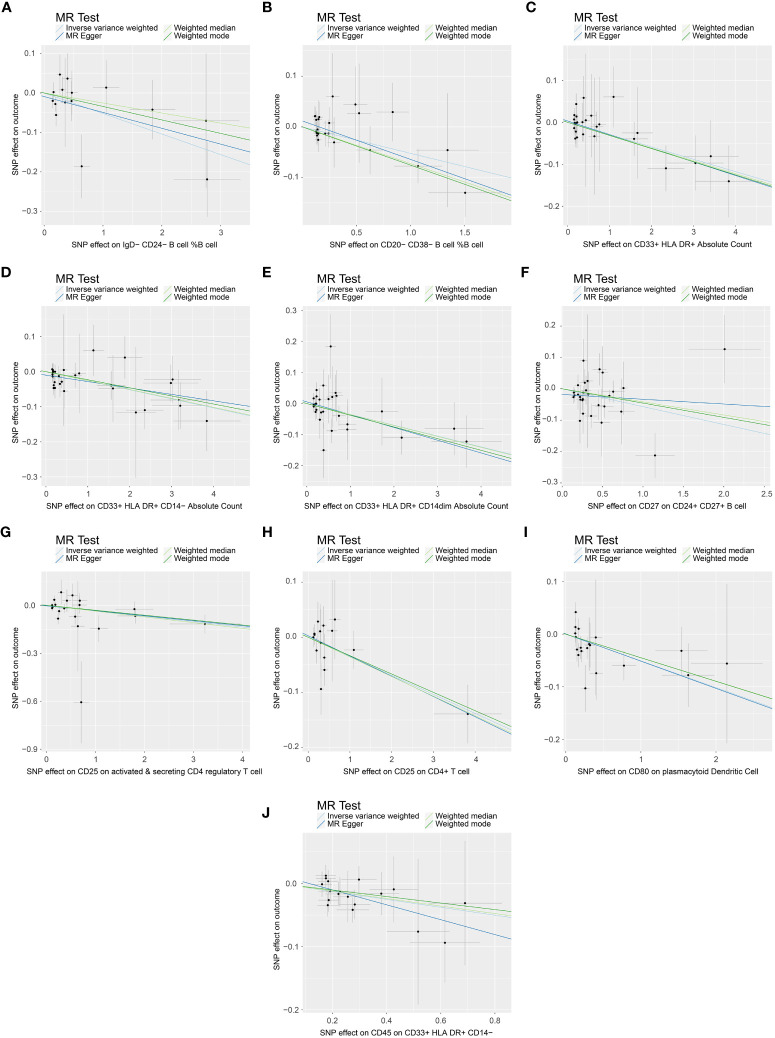
Scatter plot demonstrating the causal association between immune cell phenotypes and decreasing incidence of PE. This visual representation highlights the relationship and is annotated with relevant single nucleotide polymorphisms (SNPs). **(A)** SNP effect on IgD-CD24- B cell %B cell; **(B)** SNP effect on CD20-CD38- B cell %B cell; **(C)** SNP effect on CD33+ HLA DR+ Absolute Count; **(D)** SNP effect on CD33+ HLA DR+ CD14- Absolute Count; **(E)** SNP effect on CD33+ HLA DR+ CD14dim Absolute Count; **(F)** SNP effect on CD27 on CD24+ CD27+ B cell; **(G)** SNP effect on CD25 on active & secreting CD4 regulatory T cell; **(H)** SNP effect on CD25 on CD4+ T cell; **(I)** SNP effect on CD80 on plasmacytoid Dendritic Cell; **(J)** SNP effect on CD45 on CD33+ HLA DR+ CD14-.

**Figure 4:**
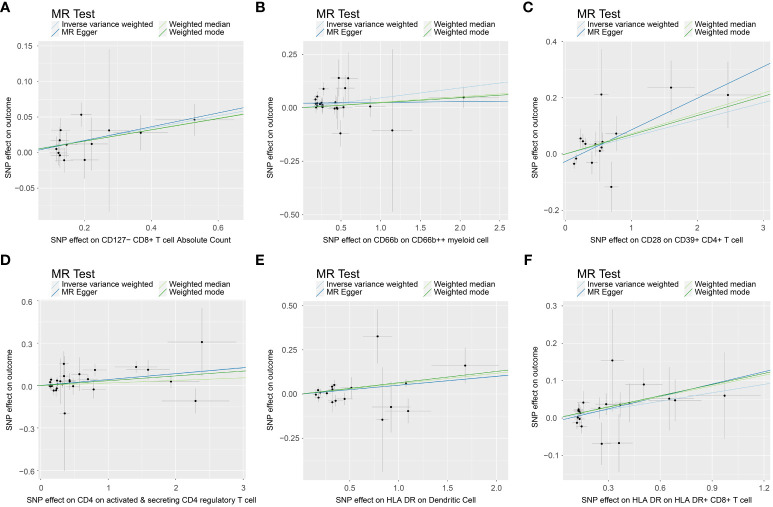
Scatter plot show casing the causal association between immune cell phenotypes and increasing incidence of PE. This graph provides a visual analysis of the association, annotated with pertinent single nucleotide polymorphisms (SNPs). **(A)** SNP effect on CD127- CD8+ T cell Absolute Count; **(B)** SNP effect on CD66b on CD66b++ myeliod cell; **(C)** SNP effect on CD28 on CD39+ CD4+ T cell; **(D)** SNP effect on CD4 on activated & secreting CD4 regulatory T cell; **(E)** SNP effect on HLA DR on Dendritic Cell; **(F)** SNP effect on HLA DR on HLA DR+ CD8+ T cell.

**Figure 5:**
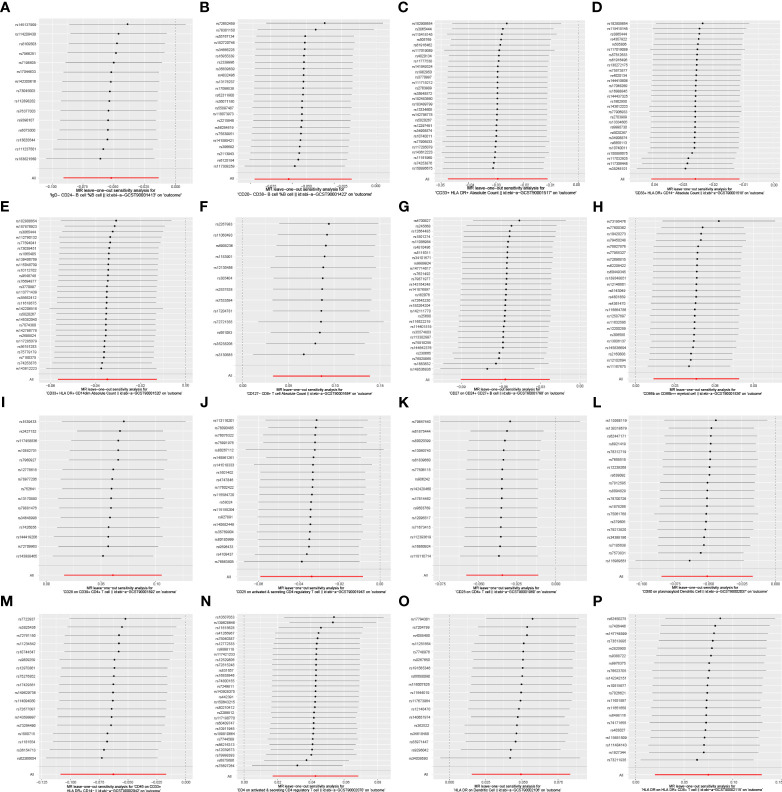
Leave-one-out analysis of the causal association between immune cell phenotypes and PE. This figure employs a leave-one-out strategy to reinforce the robustness of the causal inference. As shown: **(A)** IgD-CD24- B cell %B cell; **(B)** CD20-CD38- B cell %B cell; **(C)** CD33+ HLA DR+ Absolute Count; **(D)** CD33+ HLA DR+ CD14- Absolute Count; **(E)** CD33+ HLA DR+ CD14dim Absolute Count; **(F)** CD127- CD8+ T cell Absolute Count; **(G)** CD27 on CD24+ CD27+ B cell; **(H)** CD66b on CD66b++ myeliod cell SNP effect on CD25 on CD4+ T cell; **(I)** CD28 on CD39+ CD4+ T cell; SNP effect on CD80 on plasmacytoid Dendritic Cell; **(J)** CD25 on active & secreting CD4 regulatory T cell; **(K)** CD25 on CD4+ T cell; **(L)** CD80 on plasmacytoid Dendritic Cell; **(M)** CD45 on CD33+ HLA DR+ CD14-; **(N)** CD4 on activated & secreting CD4 regulatory T cell; **(O)** HLA DR on Dendritic Cell; **(P)** HLA DR on HLA DR+ CD8+ T cell.

In the attached document, **Supplementary Table S5** illustrates the causal links between 16 immune cell phenotypes and PE selected based on a P-value less than 0.01 according to the IVW method. This table also presents results from three additional analytic methods: MR-Egger, weighted median, and weighted mode. These methods serve as complementary analytical tools, aligning broadly with the primary IVW method findings. A forest plot (**Figure 2**) consolidates the Mendelian randomization analysis results of these 16 immune phenotypes in relation to PE. And the robustness of our findings was further confirmed in Scatter plot (**Figures 3**, **4**). Additionally, we conducted three types of sensitivity analyses to ensure the robustness of our findings: leave-one-out analysis, Cochran’ s Q test, and MR-Egger method for horizontal pleiotropy. The results of these analyses are presented in **Figure 5** (leave-one-out plot), and **Supplementary Table S6** (Cochran’ s Q test) and **Supplementary Table S7** (horizontal pleiotropy), respectively. These analyses further confirm the reliability of our results.

### Causal associations between circulating inflammatory proteins and PE

**Figure 6:**
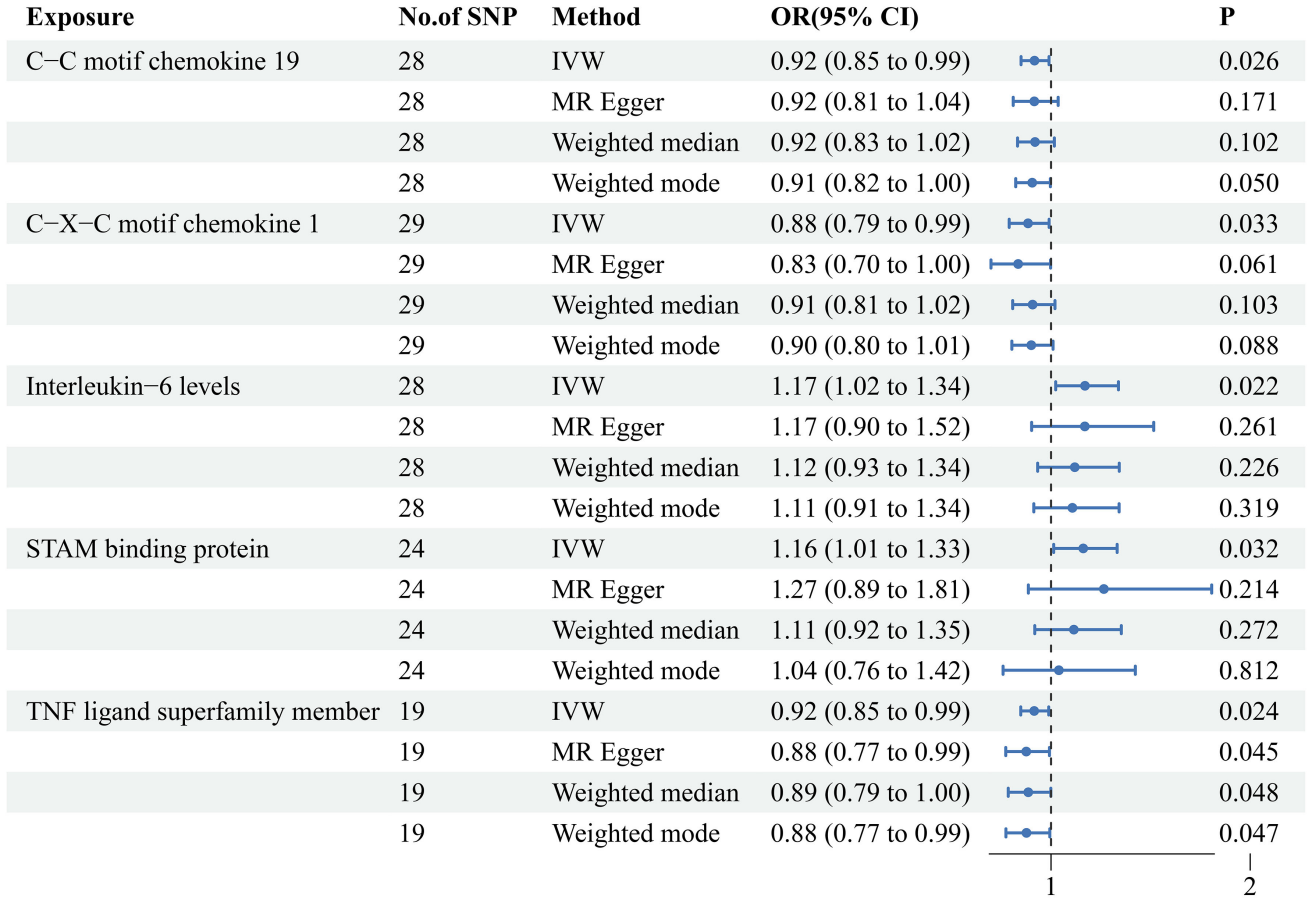
MR analysis of causal relationships between circulating inflammatory proteins and PE. Depicting the results with inverse-variance weighting (IVW), MR Egger, Weighted median and Weighted mode. This figure includes odds ratios (OR), confidence intervals (CI), and the implicated single nucleotide polymorphisms (SNPs).

**Figure 7:**
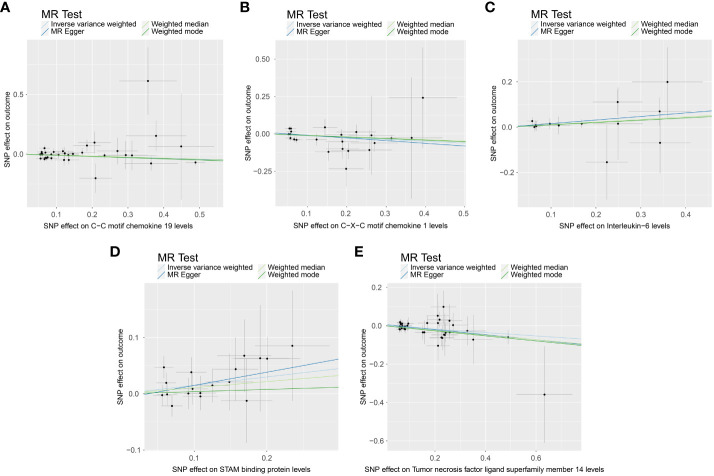
Scatter plot depicting the causal link between circulating inflammatory proteins and PE. This figure illustrates the association with a focus on the relevant single nucleotide polymorphisms (SNPs) involved. **(A)** SNP effect on C-C motif chemokine 19 levels; **(B)** SNP effect on C-X-C motif chemokine 1 level; **(C)** SNP effect on Interleukin-6 levels; **(D)** SNP effect on STAM binding protein levels; **(E)** SNP effect on Tumor necrosis factor ligand superfamily member 14 levels.

**Figure 8:**
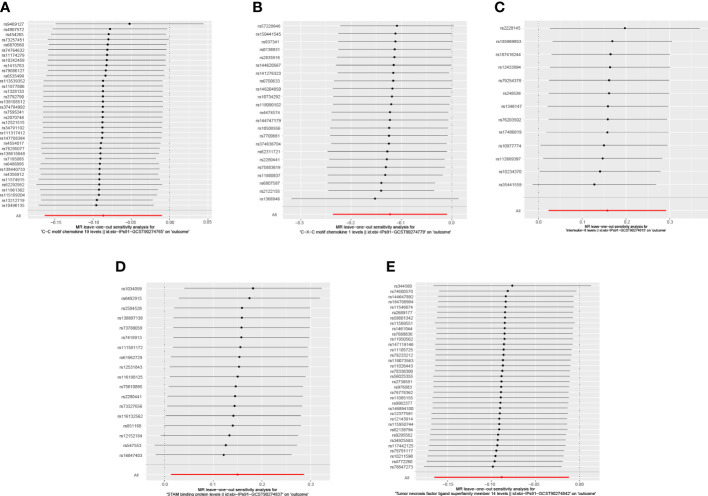
Leave-one-out analysis illustrating the causal relationship between circulating inflammatory proteins and PE. This figure demonstrates the stability of the causal inference through a leave-one-out methodology. As shown: **(A)** C-C motif chemokine 19 levels; **(B)** C-X-C motif chemokine 1 level; **(C)** Interleukin-6 levels; **(D)** STAM binding protein levels; **(E)** Tumor necrosis factor ligand superfamily member 14 levels.

When we employed the same screening criterion as that used for immune cells (p < 0.01), no significant proteins were identified. Consequently, we adopted a more lenient criterion (p < 0.05). **Supplementary Table S8** presents findings that highlight the causal connections between five circulating inflammatory proteins and PE. These proteins were identified based on a significance level of p < 0.05 in the IVW method. In a manner akin to the immune cell analysis, the table incorporates outcomes from MR-Egger, Weighted Median, and Weighted Mode methodologies. **Figure 6** (a forest plot) succinctly outlines the Mendelian randomization analysis outcomes concerning these five circulating inflammatory proteins and their association with PE. The robustness of these insights is further underscored through the depiction in a scatter plot (**Figure 7**). In addition to our primary analysis, we have reinforced the validity of our results through a comprehensive set of sensitivity analyses, encompassing a leave-one-out approach, Cochran’s Q test, and the MR-Egger method for detecting horizontal pleiotropy. The corresponding results are meticulously documented in **Figure 8** (leave-one-out analysis) and **Supplementary Table S9** (Cochran’s Q test) and **Supplementary Table S10** (horizontal pleiotropy), enhancing the robustness and reliability of our findings.

## Discussion

**Figure 9:**
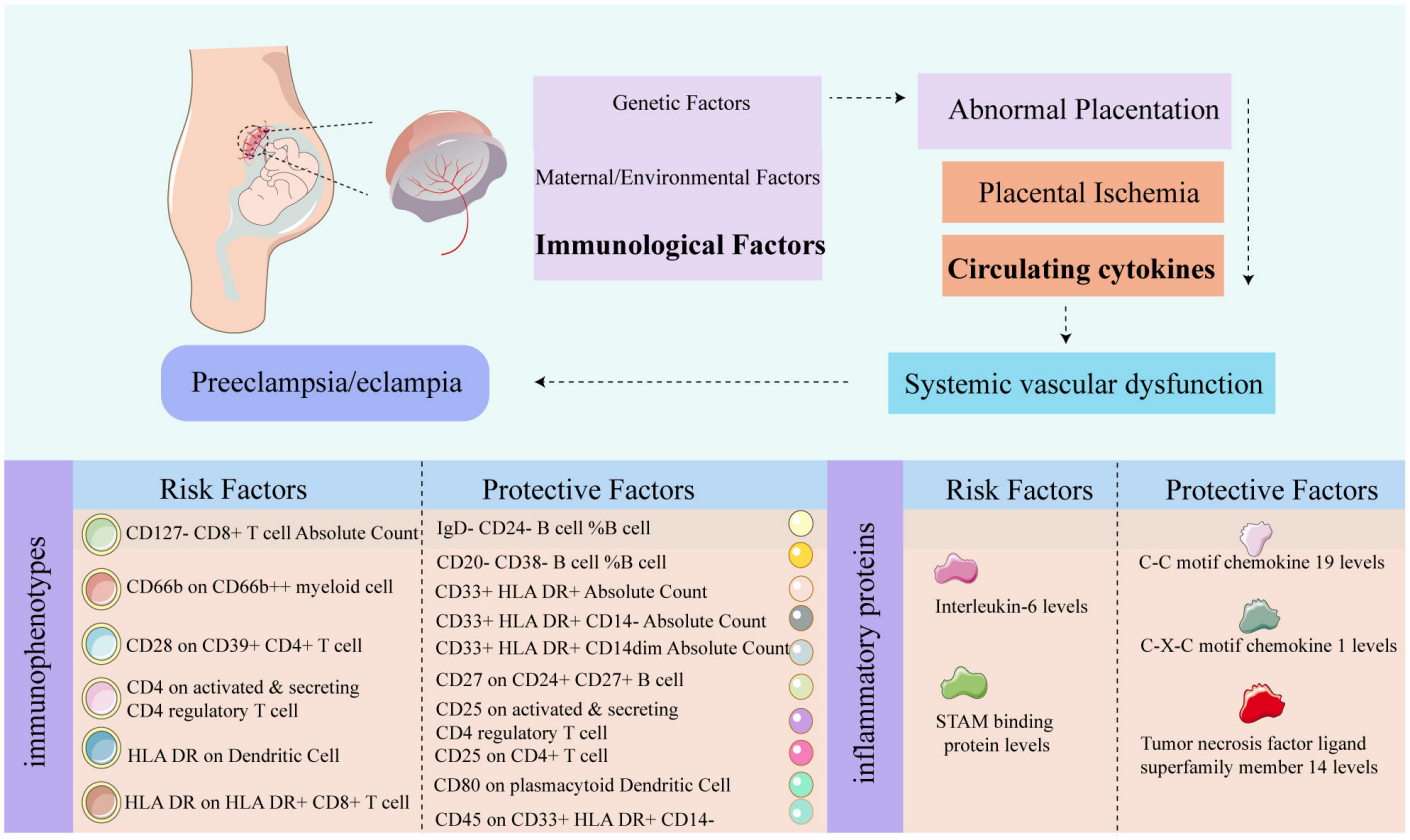
Graphic summary diagram. The relevant pathological mechanisms of preeclampsia. The risk and protective factors associated with PE in 731 immune shapes, and the risk and protective factors associated with PE in 91 inflammatory proteins.

Our research reveals that 16 immunophenotypes (p <0.01) and 5 circulating inflammatory proteins (p <0.05) exhibit causal relationships with PE by integrating extensive individual and aggregated GWAS datasets. We conducted various sensitivity analyses to assess the robustness of our findings and mitigate potential bias arising from pleiotropic effects. Notably, ten immunophenotypes phenotypes are identified as protective factors, including IgD- CD24- B cell, CD20- CD38- B cell and CD27 on CD24+ CD27+ B cell and others. Conversely, six immunophenotypes are recognized as risk factors associated with the occurrence of PE, comprising CD127- CD8+ T cell Absolute Count, CD66b on CD66b++ myeloid cell, CD28 on CD39+ CD4+ T cell and others. Meanwhile, we extend to examining the impact of 91 circulating inflammatory factors on PE. The results revealed that a total of 5 inflammatory factors are associated with PE. Among them, the levels of C-C motif chemokine 19 (CCL19), C-X-C motif chemokine 1 (CXCL1), and TNFSF14 exhibit a negative correlation with PE, acting as protective factors. Conversely, the levels of Interleukin-6 (IL-6) and STAM binding protein are identified as risk factors for the occurrence of PE. Female immune responses undergo alterations during pregnancy to maintain protective characteristics against diseases while facilitating fetal tolerance. Our results analysis reveals associations with PE involving various phenotypes of monocytes, dendritic cells, T cells and B cells. We systematically discuss these immune cell types and inflammatory factors to provide comprehensive insights into their roles in the context of PE, as shown in **Figure 9**.

### Analysis between immune cell phenotypes and PE

HLA DR expression on dendritic cells (DCs) is pivotal in regulating the balance between Th1/Th17 and Th2/Treg responses. Elevated decidual myeloid dendritic cell (mDC) levels in PE patients suggest an aberrant boost in Th1-type immune responses, implicating excessive DC maturation as a potential pathogenic factor (29). CD33 is a 67-kDa transmembrane protein that undergoes glycosylation, belonging to the sialoadhesin receptor (Siglec) family, which binds to sialic acid and is found on the cells of myeloid lineage (30). HLA-DR is an MHC-II (Major Histocompatibility Complex Class II) molecule composed of two subunits, referred to as the α-subunit and β-subunit, respectively. HLA-DR is expressed on B lymphocytes, monocytes, macrophages, activated T lymphocytes, activated NK lymphocytes, and human dendritic cells (31). The decidua contains two cell subsets: CD33(+)/HLA-DR(-) and CD33(+)/HLA-DR(+/-). Both subtypes express arginases, iNOS, IDO, and exhibit a characteristic cytokine profile. Notably, both subsets strongly inhibit T-cell proliferation, suggesting a potential significant role in promoting immune tolerance during pregnancy (32). CD33+ HLA DR+ myeloid cell mainly as antigen-presenting cell and regulate immuneIntriguingly, our results show that CD25 expression on activated and secreting CD4 regulatory T cells acts protectively against preeclampsia, while CD4 expression on these cells signifies a risk. This dichotomy underscores the importance of the activation status and surface marker expression of CD4 regulatory T cells in maintaining immune homeostasis, crucial for preventing overactive immune responses and autoimmune pathologies (33). A clinical study indicated that the frequencies of CD4+CD25+Foxp3+ and CD8+CD25+Foxp3+ cells were significantly lower in women with preeclampsia compared to healthy pregnant and non-pregnant individuals (34). CD4+CD25+ regulatory T (Treg) cells exhibit the presence of the transcription factor FoxP3, which, in collaboration with other transcription factors, intricately governs the development and functionality of this particular subset (35). CD4+CD25+ regulatory T cells express the transcription factor FoxP3, inducing the upregulation of CD25 and other molecules associated with Treg cells, such as cytotoxic T lymphocyte-associated antigen 4 and the glucocorticoid-induced tumor necrosis factor receptor. Simultaneously, they inhibit the production of interleukin-2 (IL-2), interferon-gamma (IFN-γ), and interleukin-4 (IL-4) by effector T cells (36). Multiple studies have indicated that the IL-2 signaling pathway through high-affinity IL-2 receptors (IL-2R) is crucial for the homeostasis of Treg cells (36). In normal pregnancy, CD4+ regulatory T cells interact with other immune cells, participating in the regulation of inflammation associated with implantation and contributing to an anti-inflammatory protective mechanism (37–39). However, the pro-inflammatory role of CD4 T cells, particularly in mediating oxidative stress and elevating blood pressure, is well-documented (40), implicating them in the pathophysiology of PE might through their influence on immune cell metabolic processes (41). Treg cells, expressing inhibitory markers such as CTLA-4 and CD39, exert a significant suppressive function (42). Studies have reported lower proportions of CD39+ CD4+ T cells in the preeclampsia group compared to normal pregnancies (43). Despite prior associations of this cell subtype with immune suppression in basic research (44), our findings indicate its correlation with preeclampsia risk. CD28, as the inaugural member of the costimulatory molecule subfamily, is characterized by its extracellular variable immunoglobulin-like domain. CD28 orchestrates phosphorylation, transcription signal transduction, metabolism, and the generation of crucial cytokines, chemokines, and survival signals, critically influencing the long-term proliferation and differentiation of T cells. The CD28 signal, depending on the cell type and context, not only mediates immune suppression by inhibiting regulatory T (Treg) cells but also, conversely, promotes Treg cells to prevent the occurrence of autoimmune diseases (45, 46). Our analysis identifies CD28 expression on CD39+ CD4+ T cells as a risk factor in PE, a finding that invites further exploration given the limited research in this area. CD127, or the alpha subunit of the IL-7 receptor, distinguishes regulatory T cells from activated T cells, predominantly marking mature T cells and regulating their proliferation and differentiation (47). CD127- CD8+ T cell Absolute Count, belonging to immature T cells, an increased abundance of this cell type may lead to the occurrence of PE.

According to the expression of CD45R/B220, pre-pro-B cells, pro-B cells, and pre-B cells can be preliminarily distinguished, further determining their differentiation based on distinct CD24 expression patterns. CD24+ CD27+ B cells, a regulatory B cell subset, are primarily involved in IL-10 production and suppressing CD4+ T cell proliferation, thus inhibiting IFN-γ/IL-17 generation (48). A clinical study indicated a negative correlation between regulatory B cells and the occurrence of PE (49), consistent with the observed results in our analysis. Immature B cells, characterized by CD20- CD38-,contributing to clone clearance and the establishment of self-immune tolerance. Therefore, CD20- CD38- B cells are present as a protective factor for PE.

### Analysis between circulating inflammatory proteins and PE

In the realm of PE prediction, the role of inflammatory factors is of paramount clinical relevance. IL-6, a widely recognized inflammatory cytokine (50), actively participates in regulating immune and inflammatory responses across various cell types at the maternal-fetal interface. *In vitro* experiments suggest that elevated local IL-6 levels may promote trophoblast functionality (51), positing a potential link to PE’s pathogenesis. Moving to STAM, it is crucial for its interactions with JAK3 and JAK2 tyrosine kinases, undergoing phosphorylation upon stimulation by cytokines such as IL-2 and GM-CSF (52). The JAK–STAT pathway, involving JAK3 and JAK2, has been implicated in the mechanisms leading to PE (53), aligning with our findings. Our study suggests CCL19 as a protective factor against PE, highlighting its role in promoting trophoblast migration and invasion via the CCR7-mediated pathway (54). Shifting focus to CXCL1, a member of the G protein-coupled receptor family (55, 56), it binds specifically to the CXC chemokine receptor 2. CXCL1 may influence the dynamics of trophoblast and endothelial cells in placental vascular formation, thus playing a role in normal pregnancy progression (57). Concluding with TNFSF14, also known as LIGHT, serving as a costimulatory molecule linked to T lymphocyte activation (58). And it is elevated in the circulation and placentas of PE patients (59),TNFSF14 is known to promote epithelial-mesenchymal transition (EMT) through pathways such as TGFβ or Erk1/2 pathways (60), processes that are critical in extra-villous trophoblast and might have relationship with PE (61). In an *in vitro* experiment using a primary syncytiotrophoblast cell model, LIGHT was found to directly induce the expression of sFlt-1 in trophoblast cells, suggesting it may be one of the pathological mechanisms underlying early-onset preeclampsia (62). Another animal experiment indicated that injecting TNFSF14 into pregnant mice induced phenotypic characteristics similar to preeclampsia (59). Our MR analysis, from a genetic perspective, substantiates the association of TNFSF14 with PE, suggesting its potential involvement in the pathophysiological processes. This provides novel insights into its role in maternal and fetal health.

### Advantages and limitations

Our analytical delves into the genetic correlations between these immune components and the risk of PE, underscoring the necessity of considering gestational age and the intricacies of the maternal-fetal microenvironment. Such an approach not only paves the way for foundational research but also promises to refine disease prediction strategies, ultimately enhancing maternal-fetal health and contributing to societal well-being. However, there are also some limitations in our study. In the GWAS dataset for immune phenotypes and inflammatory factors, no gender-based stratification was performed. As our study exclusively encompasses the female population, this introduces a potential bias. A notable limitation is the identification of 16 immunophenotypes and 5 inflammatory proteins associated with PE, which raises the possibility of false positives given the relatively high number of positive results. Consequently, further clinical validation is essential to substantiate these associations. Another concern is the potential influence of pregnancy on the body’s immune status. Although our analysis utilized the steiger-test method to mitigate reverse causation, the complete elimination of such causality cannot be definitively assured.

This highlights the need for continued vigilance in interpreting the findings and underscores the importance of further research to unravel the complex interactions between pregnancy, immune system dynamics, and the development of PE. Our study underscores the crucial contributions of various immune cell types and circulating inflammatory proteins in the development of PE. This understanding may open up new avenues for innovative approaches to address PE, ultimately improving the health outcomes for both mothers and newborns.

## Data availability statement

The original contributions presented in the study are included in the article/**Supplementary Material**. Further inquiries can be directed to the corresponding authors.

## Ethics statement

We used summary-level data from publicly available GWAS studies which have received ethical approval from their respective institutional review boards and informed consent from all participants. Additionally, since this research does not involve any personal data, it is not necessary to apply for new ethical review approval. No administrative permissions were required to access the data.

## Author contributions

YL: Data curation, Investigation, Writing – original draft, Writing – review & editing. YZ: Methodology, Writing – original draft, Writing – review & editing. LD: Funding acquisition, Investigation, Supervision, Writing – review & editing. DC: Funding acquisition, Investigation, Resources, Supervision, Writing – review & editing.
