# Supplementary material for: The genetic relationships between immune cell traits, circulating inflammatory proteins and preeclampsia/eclampsia
Source: Front Immunol. 2024 May 30;15:1389843. doi: 10.3389/fimmu.2024.1389843 (PMC11170637; doi:10.3389/fimmu.2024.1389843)
Supplement: Supplementary file 11 [file Table_1.docx]

**STROBE-MR checklist of recommended items to address in reports of Mendelian randomization studies**^1^ ^2^

| **Item No.** | **Section** | **Checklist item** | **Page No.** | **Relevant text from manuscript** |
| --- | --- | --- | --- | --- |
| 1 | **TITLE and ABSTRACT** | Indicate Mendelian randomization (MR) as the study’s design in the title and/or the abstract if that is a main purpose of the study |  | Our study aimed to employ a two-sample mendelian randomization (MR) analysis to assess the potential causal effects of immune cell phenotypes and circulating inflammatory proteins on the onset of PE. |
|  | **INTRODUCTION** |  |  |  |
| 2 | **Background** | Explain the scientific background and rationale for the reported study. What is the exposure? Is a potential causal relationship between exposure and outcome plausible? Justify why MR is a helpful method to address the study question |  | Particularly noteworthy is the role of immune dysregulation and inflammation, which are now understood as key contributors to placental dysfunction, leading to the manifestation of preeclampsia  In our study, we apply two-sample MR analysis to deepen our understanding of the causal implications of peripheral immunity in the risk of PE, providing new insights into potential therapeutic strategies and preventive measures.  mendelian randomization (MR) presents an innovative method, which leverages genetic variants as instrumental variables to estimate the causal association between exposure and disease outcome |
| 3 | **Objectives** | State specific objectives clearly, including pre-specified causal hypotheses (if any). State that MR is a method that, under specific assumptions, intends to estimate causal effects |  | In our study, we apply two-sample MR analysis to deepen our understanding of the causal implications of peripheral immunity in the risk of PE, providing new insights into potential therapeutic strategies and preventive measures. |
|  | **METHODS** |  |  |  |
| 4 | **Study design and data sources** | Present key elements of the study design early in the article. Consider including a table listing sources of data for all phases of the study. For each data source contributing to the analysis, describe the following: |  |  |
|  | a) | Setting: Describe the study design and the underlying population, if possible. Describe the setting, locations, and relevant dates, including periods of recruitment, exposure, follow-up, and data collection, when available. |  | The design and progression of our study are encapsulated in Fig.1. |
|  | b) | Participants: Give the eligibility criteria, and the sources and methods of selection of participants. Report the sample size, and whether any power or sample size calculations were carried out prior to the main analysis |  | The initial GWAS analyzed a dataset that included 3,757 individuals of European descent, with a careful design to avoid any overlap between cohorts.  ”，“Data on 91 circulating inflammatory proteins were derived from a GWAS conducted on 14,824 participants of European ancestry |
|  | c) | Describe measurement, quality control and selection of genetic variants |  | The significance level for selecting IVs for each immune trait was set at 1×10^−5^. |
|  | d) | For each exposure, outcome, and other relevant variables, describe methods of assessment and diagnostic criteria for diseases |  | These encompass 118 absolute cell (AC) counts, 389 median fluorescence intensities (MFI), 32 morphological parameters (MP), and 192 relative cell (RC) counts[20]. Covering an extensive range of immune cells, this dataset included B cells, dendritic cells (DCs), T cells at various maturity stages, monocytes, myeloid cells, and TBNK (T cells, B cells, natural killer cells) and Treg panels, with MPs specifically focusing on DCs and TBNK panels. |
|  | e) | Provide details of ethics committee approval and participant informed consent, if relevant |  | For these original GWAS studies, corresponding ethical approvals have been obtained. Additionally, since this research does not involve any personal data, it is not necessary to apply for new ethical review approval. |
| 5 | **Assumptions** | Explicitly state the three core IV assumptions for the main analysis (relevance, independence and exclusion restriction) as well assumptions for any additional or sensitivity analysis |  | Firstly, the Relevance Assumption posits that genetic variants utilized as instrumental variables must demonstrate a significant and robust association with the exposure under investigation. This foundational premise ensures that the genetic instruments are effectively linked to the exposure, thereby facilitating a valid examination of the exposure-outcome relationship. Secondly, the Independence Assumption mandates that these genetic variants remain uninfluenced by any confounders, underpinning the concept that the allocation of genetic variants occurs in a manner akin to random assignment at conception, thus safeguarding against confounding biases. Lastly, the Exclusion Restriction necessitates that the influence of genetic variants on the outcome is mediated exclusively through the exposure, precluding any alternative pathways. |
| 6 | **Statistical methods: main analysis** | Describe statistical methods and statistics used |  |  |
|  | a) | Describe how quantitative variables were handled in the analyses (i.e., scale, units, model) |  | NA |
|  | b) | Describe how genetic variants were handled in the analyses and, if applicable, how their weights were selected |  | The significance level for selecting IVs for each immune trait was set at 1×10^−5^. The linkage disequilibrium (LD) pruning of these SNPs, where SNPs were pruned based on an LD r^2^ threshold of less than 0.001 within a 10000 kb distance. For the selection of IVs for circulating inflammatory proteins, the significance level was set at 1×10^−5^, consistent with the approach for immune traits. SNPs were pruned based on an LD r^2^ threshold of less than 0.001 within a 10000 kb distance. For the outcome PE the significance level for IV selection was adjusted to 5×10^-5^. |
|  | c) | Describe the MR estimator (e.g. two-stage least squares, Wald ratio) and related statistics. Detail the included covariates and, in case of two-sample MR, whether the same covariate set was used for adjustment in the two samples |  | Our primary analysis utilized the inverse variance-weighted (IVW) method, a well-established approach in MR studies. |
|  | d) | Explain how missing data were addressed |  | NA |
|  | e) | If applicable, indicate how multiple testing was addressed |  |  |
| 7 | **Assessment of assumptions** | Describe any methods or prior knowledge used to assess the assumptions or justify their validity |  | NA |
| 8 | **Sensitivity analyses and additional analyses** | Describe any sensitivity analyses or additional analyses performed (e.g. comparison of effect estimates from different approaches, independent replication, bias analytic techniques, validation of instruments, simulations) |  | To enhance result robustness, we conducted supplementary analyses using the MR-Egger regression methods[24], weighted median[25] and weighted mode[26].We assessed potential directional pleiotropy by examining the intercept value in the MR-Egger regression[27]. Heterogeneity was evaluated using Cochran’s Q test . |
| 9 | **Software and pre-registration** |  |  |  |
|  | a) | Name statistical software and package(s), including version and settings used |  | All statistical analyses and data visualisation were performed using R software, version 4.3.1. For mendelian randomization, we utilized the"TwoSampleMR","MendelianRandomization" package in R, which facilitated dataset harmonization and the implementation of various MR methods. |
|  | b) | State whether the study protocol and details were pre-registered (as well as when and where) |  | NA |
|  | **RESULTS** |  |  |  |
| 10 | **Descriptive data** |  |  |  |
|  | a) | Report the numbers of individuals at each stage of included studies and reasons for exclusion. Consider use of a flow diagram |  | This data was part of their 9th release of GWAS summaries, which was made available on May 11, 2023. The dataset included 1413 cases and 287137 controls. |
|  | b) | Report summary statistics for phenotypic exposure(s), outcome(s), and other relevant variables (e.g. means, SDs, proportions) |  | NA |
|  | c) | If the data sources include meta-analyses of previous studies, provide the assessments of heterogeneity across these studies |  | NA |
|  | d) | For two-sample MR:  i.  Provide justification of the similarity of the genetic variant-exposure associations between the exposure and outcome samples  ii.  Provide information on the number of individuals who overlap between the exposure and outcome studies |  | NA |
| 11 | **Main results** |  |  |  |
|  | a) | Report the associations between genetic variant and exposure, and between genetic variant and outcome, preferably on an interpretable scale |  | we pinpointed six immunophenotypes as risk factors for PE: CD4 on activated and secreting CD4 regulatory T cells (Treg panel), CD28 on CD39+ CD4+ T cells (Treg panel), CD127- CD8+ T cell AC (Treg panel), HLA DR on HLA DR+ CD8+ T cell (TBNK panel), CD66b on CD66b++ myeloid cells (Myeloid cell panel), and HLA DR on dendritic cells (cDC panel). Conversely, we identified ten immunophenotypes that appear to have protective effects against PE. These include CD45 on CD33br HLA DR+ CD14- (Myeloid cell panel), CD33+ HLA DR+ AC (Myeloid cell panel), CD33+ HLA DR+ CD14- AC (Myeloid cell panel), CD33+ HLA DR+ CD14dim AC (Myeloid cell panel), CD27 on CD24+ CD27+ B cell (B cell panel), CD20- CD38- %B cell (B cell panel), IgD- CD24- %B cell (B cell panel), CD80 on plasmacytoid DC (cDC panel), CD25 on CD4+ T cell (Treg panel), and CD25 on activated & secreting CD4 regulatory T cell (Treg panel). |
|  | b) | Report MR estimates of the relationship between exposure and outcome, and the measures of uncertainty from the MR analysis, on an interpretable scale, such as odds ratio or relative risk per SD difference |  | we pinpointed six immunophenotypes as risk factors for PE: CD4 on activated and secreting CD4 regulatory T cells (Treg panel), CD28 on CD39+ CD4+ T cells (Treg panel), CD127- CD8+ T cell AC (Treg panel), HLA DR on HLA DR+ CD8+ T cell (TBNK panel), CD66b on CD66b++ myeloid cells (Myeloid cell panel), and HLA DR on dendritic cells (cDC panel). Conversely, we identified ten immunophenotypes that appear to have protective effects against PE. These include CD45 on CD33br HLA DR+ CD14- (Myeloid cell panel), CD33+ HLA DR+ AC (Myeloid cell panel), CD33+ HLA DR+ CD14- AC (Myeloid cell panel), CD33+ HLA DR+ CD14dim AC (Myeloid cell panel), CD27 on CD24+ CD27+ B cell (B cell panel), CD20- CD38- %B cell (B cell panel), IgD- CD24- %B cell (B cell panel), CD80 on plasmacytoid DC (cDC panel), CD25 on CD4+ T cell (Treg panel), and CD25 on activated & secreting CD4 regulatory T cell (Treg panel). |
|  | c) | If relevant, consider translating estimates of relative risk into absolute risk for a meaningful time period |  | NA |
|  | d) | Consider plots to visualize results (e.g. forest plot, scatterplot of associations between genetic variants and outcome versus between genetic variants and exposure) |  | FIG.2,FIG.3,FIG.4 |
| 12 | **Assessment of assumptions** |  |  |  |
|  | a) | Report the assessment of the validity of the assumptions |  | To address the issue of weak instrumental variables, we estimated the F-statistic for each genetic variant used as an instrument. SNPs with an F-statistic below 10 were considered weak and subsequently excluded from our analysis, ensuring the robustness of our findings. |
|  | b) | Report any additional statistics (e.g., assessments of heterogeneity across genetic variants, such as *I^2^*, Q statistic or E-value) |  | Heterogeneity was evaluated using Cochran’s Q test |
| 13 | **Sensitivity analyses and additional analyses** |  |  |  |
|  | a) | Report any sensitivity analyses to assess the robustness of the main results to violations of the assumptions |  | To enhance result robustness, we conducted supplementary analyses using the MR-Egger regression methods[24], weighted median[25] and weighted mode[26].We assessed potential directional pleiotropy by examining the intercept value in the MR-Egger regression[27]. Heterogeneity was evaluated using Cochran’s Q test[28] |
|  | b) | Report results from other sensitivity analyses or additional analyses |  | To enhance result robustness, we conducted supplementary analyses using the MR-Egger regression methods[24], weighted median[25] and weighted mode[26].We assessed potential directional pleiotropy by examining the intercept value in the MR-Egger regression[27]. Heterogeneity was evaluated using Cochran’s Q test[28] |
|  | c) | Report any assessment of direction of causal relationship (e.g., bidirectional MR) |  | NA |
|  | d) | When relevant, report and compare with estimates from non-MR analyses |  | NA |
|  | e) | Consider additional plots to visualize results (e.g., leave-one-out analyses) |  | FIG.5 |
|  | **DISCUSSION** |  |  |  |
| 14 | **Key results** | Summarize key results with reference to study objectives |  | we pinpointed six immunophenotypes as risk factors for PE: CD4 on activated and secreting CD4 regulatory T cells (Treg panel), CD28 on CD39+ CD4+ T cells (Treg panel), CD127- CD8+ T cell AC (Treg panel), HLA DR on HLA DR+ CD8+ T cell (TBNK panel), CD66b on CD66b++ myeloid cells (Myeloid cell panel), and HLA DR on dendritic cells (cDC panel). Conversely, we identified ten immunophenotypes that appear to have protective effects against PE. These include CD45 on CD33br HLA DR+ CD14- (Myeloid cell panel), CD33+ HLA DR+ AC (Myeloid cell panel), CD33+ HLA DR+ CD14- AC (Myeloid cell panel), CD33+ HLA DR+ CD14dim AC (Myeloid cell panel), CD27 on CD24+ CD27+ B cell (B cell panel), CD20- CD38- %B cell (B cell panel), IgD- CD24- %B cell (B cell panel), CD80 on plasmacytoid DC (cDC panel), CD25 on CD4+ T cell (Treg panel), and CD25 on activated & secreting CD4 regulatory T cell (Treg panel). |
| 15 | **Limitations** | Discuss limitations of the study, taking into account the validity of the IV assumptions, other sources of potential bias, and imprecision. Discuss both direction and magnitude of any potential bias and any efforts to address them |  | However, there are also some limitations in our study. In the GWAS dataset for immune phenotypes and inflammatory factors, no gender-based stratification was performed. As our study exclusively encompasses the female population, this introduces a potential bias. A notable limitation is the identification of 16 immunophenotypes and 5 inflammatory proteins associated with PE, which raises the possibility of false positives given the relatively high number of positive results. Consequently, further clinical validation is essential to substantiate these associations. Another concern is the potential influence of pregnancy on the body's immune status. Although our analysis utilized the steiger-test method to mitigate reverse causation, the complete elimination of such causality cannot be definitively assured. |
| 16 | **Interpretation** |  |  |  |
|  | a) | Meaning: Give a cautious overall interpretation of results in the context of their limitations and in comparison with other studies |  | A clinical study indicated a negative correlation between regulatory B cells and the occurrence of PE[49] , consistent with the observed results in our analysis. Immature B cells, characterized by CD20- CD38-,contributing to clone clearance and the establishment of self-immune tolerance. Therefore, CD20- CD38- B cells are present as a protective factor for PE |
|  | b) | Mechanism: Discuss underlying biological mechanisms that could drive a potential causal relationship between the investigated exposure and the outcome, and whether the gene-environment equivalence assumption is reasonable. Use causal language carefully, clarifying that IV estimates may provide causal effects only under certain assumptions |  | our findings indicate its correlation with preeclampsia risk. CD28, as the inaugural member of the costimulatory molecule subfamily, is characterized by its extracellular variable immunoglobulin-like domain. CD28 orchestrates phosphorylation, transcription signal transduction, metabolism, and the generation of crucial cytokines, chemokines, and survival signals, critically influencing the long-term proliferation and differentiation of T cells. The CD28 signal, depending on the cell type and context, not only mediates immune suppression by inhibiting regulatory T (Treg) cells but also, conversely, promotes Treg cells to prevent the occurrence of autoimmune diseases |
|  | c) | Clinical relevance: Discuss whether the results have clinical or public policy relevance, and to what extent they inform effect sizes of possible interventions |  | Our MR analysis, from a genetic perspective, substantiates the association of TNFSF14 with PE, suggesting its potential involvement in the pathophysiological processes. This provides novel insights into its role in maternal and fetal health. |
| 17 | **Generalizability** | Discuss the generalizability of the study results (a) to other populations, (b) across other exposure periods/timings, and (c) across other levels of exposure |  | no gender-based stratification was performed. As our study exclusively encompasses the female population, this introduces a potential bias. |
|  | **OTHER INFORMATION** |  |  |  |
| 18 | **Funding** | Describe sources of funding and the role of funders in the present study and, if applicable, sources of funding for the databases and original study or studies on which the present study is based |  | Our work was supported by grants from the National Natural Science Foundation of China (No. 82171666, 81830045, 82071652,), National Key R&D Program of China (No.2022YFC2704500, 2022YFC2704505). |
| 19 | **Data and data sharing** | Provide the data used to perform all analyses or report where and how the data can be accessed, and reference these sources in the article. Provide the statistical code needed to reproduce the results in the article, or report whether the code is publicly accessible and if so, where |  | For these original GWAS studies, corresponding ethical approvals have been obtained. Additionally, since this research does not involve any personal data, it is not necessary to apply for new ethical review approval. |
| 20 | **Conflicts of Interest** | All authors should declare all potential conflicts of interest |  | The authors affirm that the study was carried out without any commercial or financial affiliations that might be perceived as potential conflicts of interest.  This study conducts a secondary analysis based on publicly available datasets. |

This checklist is copyrighted by the Equator Network under the Creative Commons Attribution 3.0 Unported (CC BY 3.0) license.

1. Skrivankova VW, Richmond RC, Woolf BAR, Yarmolinsky J, Davies NM, Swanson SA, et al. Strengthening the Reporting of Observational Studies in Epidemiology using Mendelian Randomization (STROBE-MR) Statement. JAMA. 2021;under review.

2. Skrivankova VW, Richmond RC, Woolf BAR, Davies NM, Swanson SA, VanderWeele TJ, et al. Strengthening the Reporting of Observational Studies in Epidemiology using Mendelian Randomisation (STROBE-MR): Explanation and Elaboration. BMJ. 2021;375:n2233.
